# Supplementary figures and images for: MUSiCC: a marker genes based framework for metagenomic normalization and accurate profiling of gene abundances in the microbiome
Source: Genome Biol. 2015 Mar 25;16(1):53. doi: 10.1186/s13059-015-0610-8 (PMC4391136; doi:10.1186/s13059-015-0610-8)

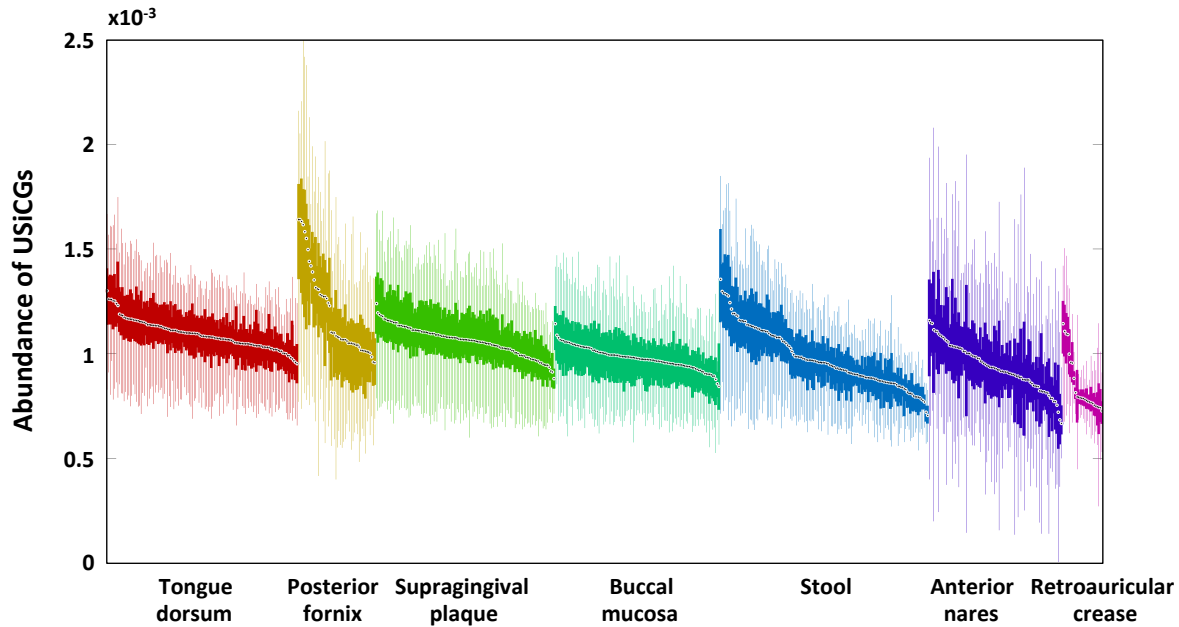

Supplement: Additional file 2: Figure S1. — Spurious inter-sample variation across HMP metagenomic samples in various body sites. See Figure 1B for definition of box and whisker plot. [file 13059_2015_610_MOESM2_ESM.pdf]

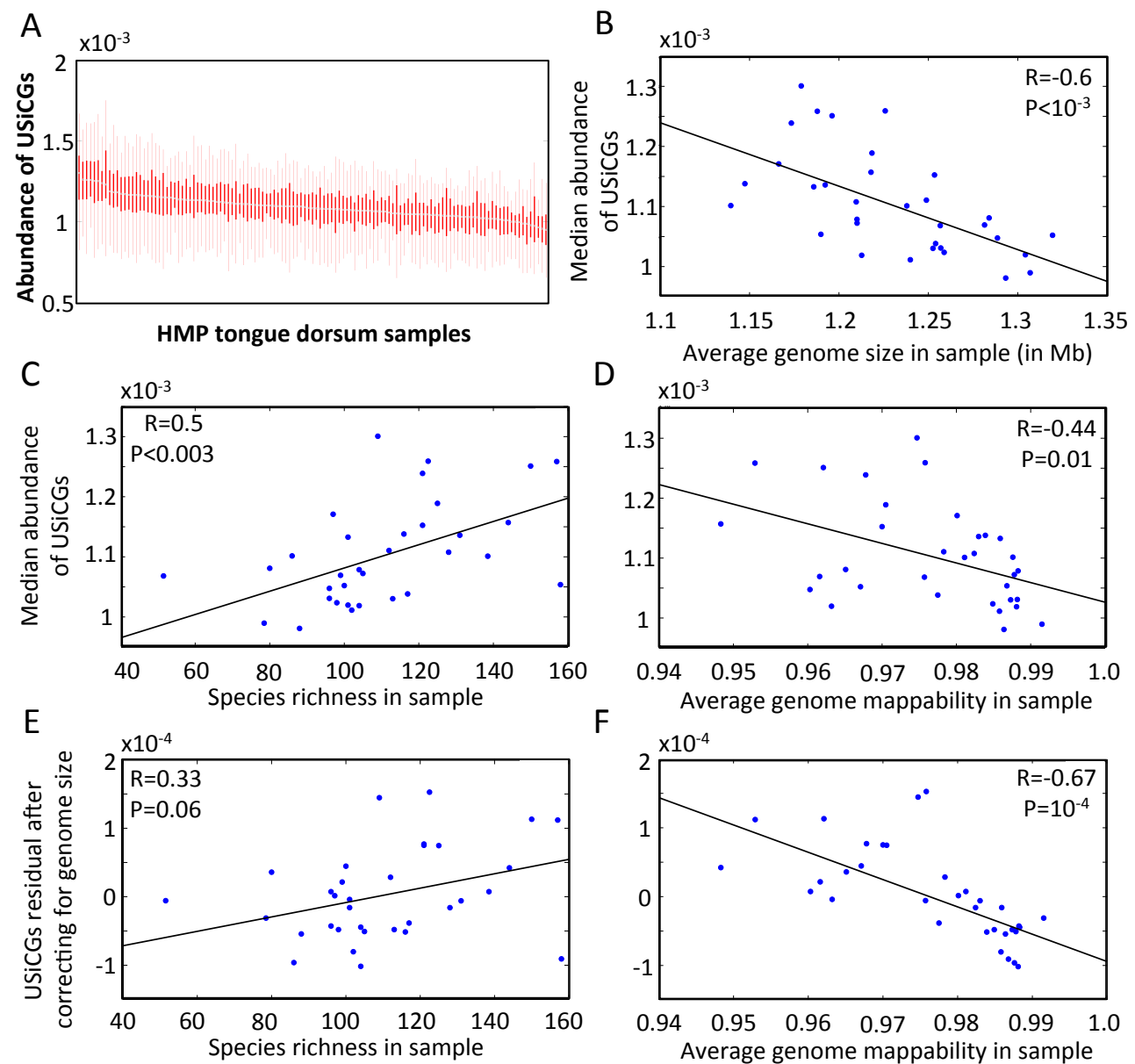

Supplement: Additional file 3: Figure S2. — Spurious inter-sample variation in HMP tongue dorsum samples is correlated with sample-specific properties. (A) The relative abundance of USiCGs across HMP tongue dorsum samples. See Figure 1B for definition of box and whisker plot. The median abundance of USiCGs across tongue dorsum samples is correlated with (B) the average genome size; R = -0.6, P <10-3, (C) the species richness in the sample; R = 0.43, P <10-3, and (D) the average genome mappability; R = -0.85, P <10-17. See Methods for more details on estimating sample-specific properties. (E, F) The correlations with species richness and genome mappability still hold after correcting the median USiCGs abundance with respect to the average genome size and using the residuals (R = 0.33, P = 0.06 and R = -0.67, P <10-4, respectively). Regression lines are illustrated in black. [file 13059_2015_610_MOESM3_ESM.pdf]

**A**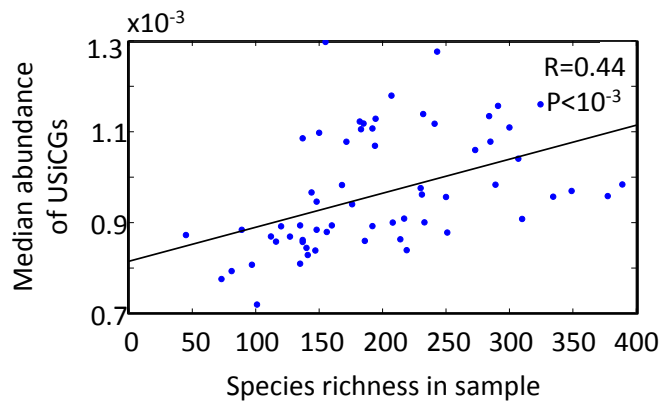**B**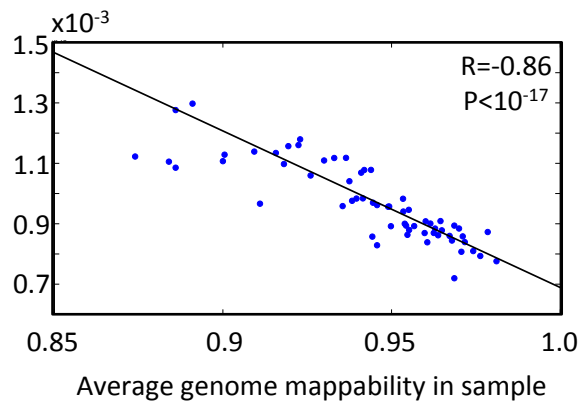**C**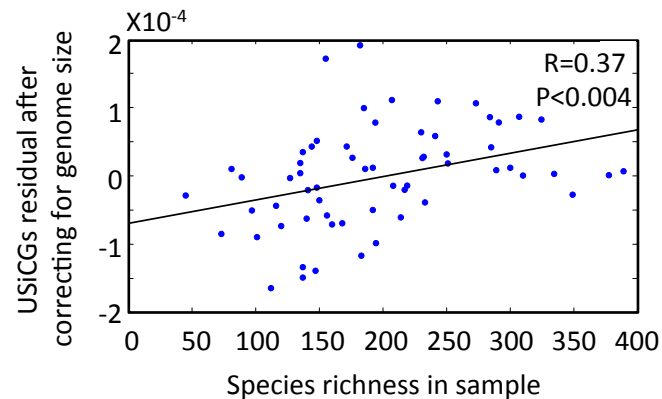**D**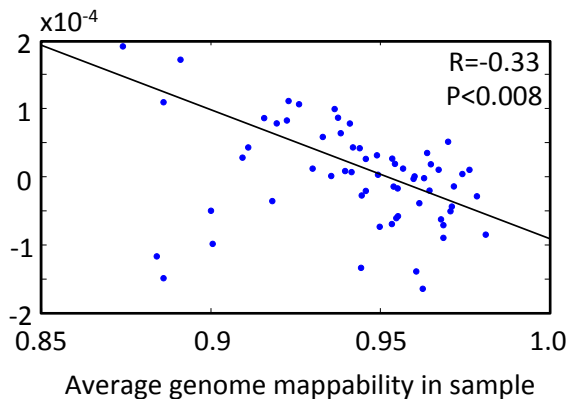

Supplement: Additional file 4: Figure S3. — Spurious variation in the abundance of USiCGs across HMP stool samples is correlated with (A) the species richness in the sample; R = 0.44, P <10-3, and with (B) the average genome mappability; R = -0.85, P <10-17. (C, D) These correlations still hold after correcting the median USiCGs abundance and using the residuals with respect to the average genome size (R = 0.35, P <0.005, and R = -0.33, P <0.008, for species richness and average genome mappability, respectively). Each point represents a single stool sample. Regression lines are illustrated in black. See Methods for more details on estimating average genome size, species richness, and genome mappability. [file 13059_2015_610_MOESM4_ESM.pdf]

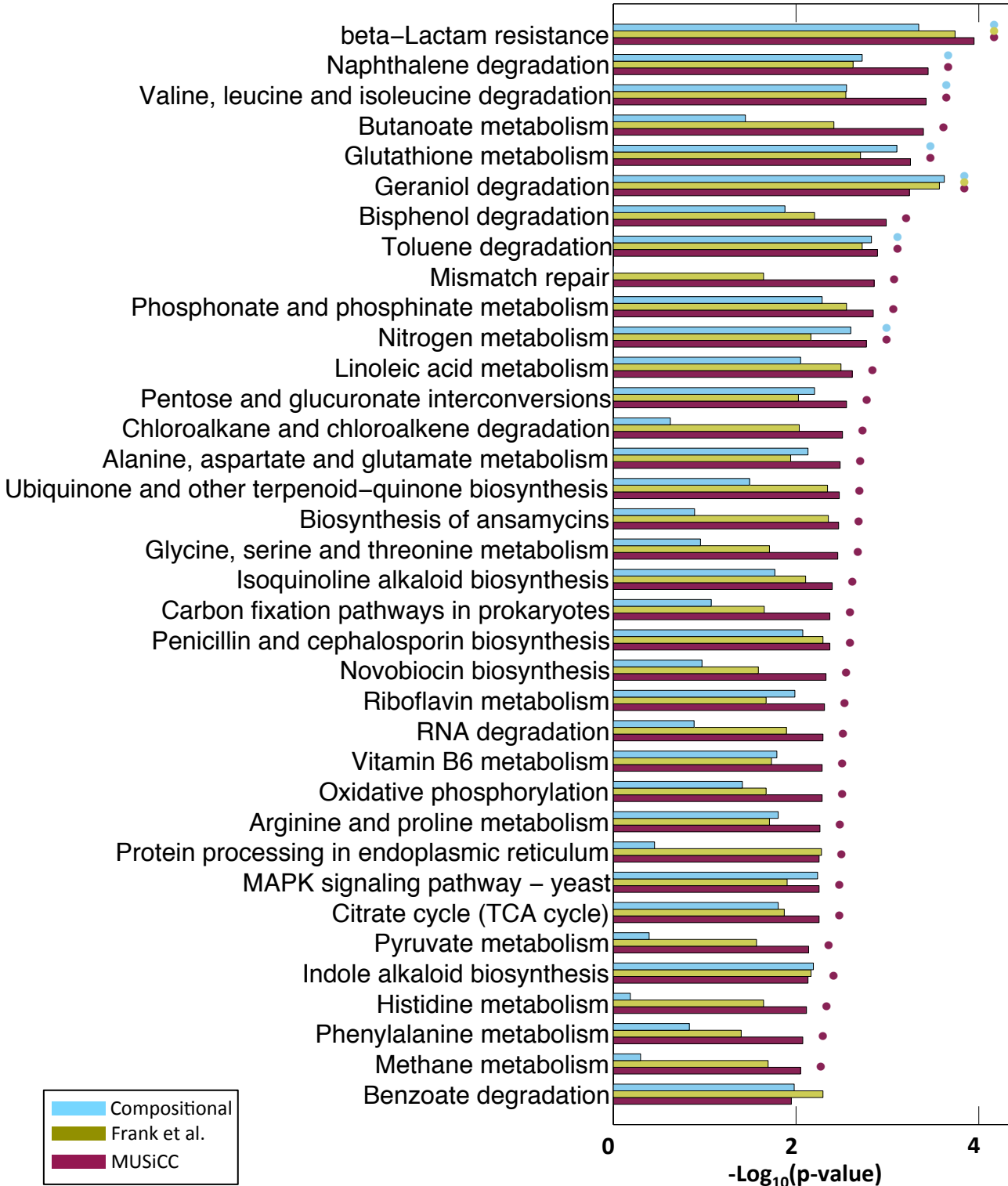

Supplement: Additional file 9: Figure S5. — Comparing the impact of standard compositional normalization, an alternative normalization approach [19], and MUSiCC on the discovery of disease-associated pathways. Pathways identified to be associated with inflammatory bowel disease using any of these three methods are illustrated. Bars denote the significance level of the association. The dots to the right of each bar indicate whether this association reached significance with FDR <0.05 with compositional normalization (cyan), the alternative normalization approach [19] (green), or MUSiCC (maroon). [file 13059_2015_610_MOESM9_ESM.pdf]

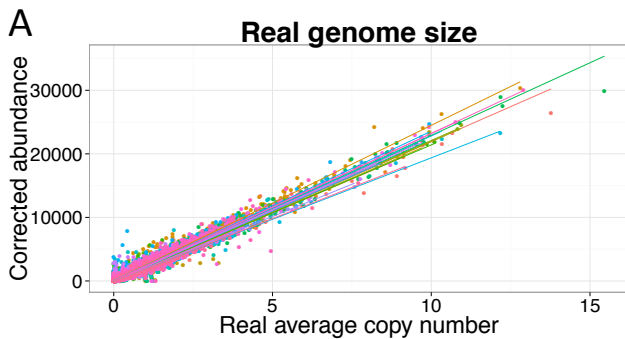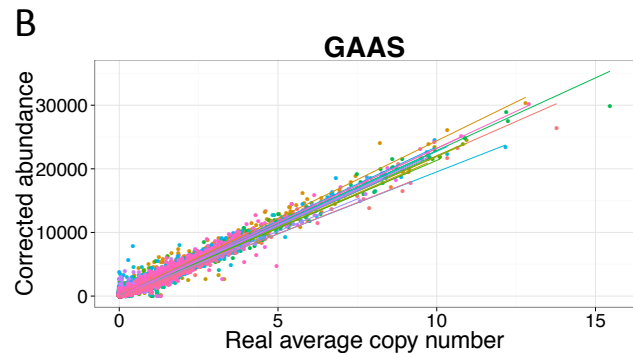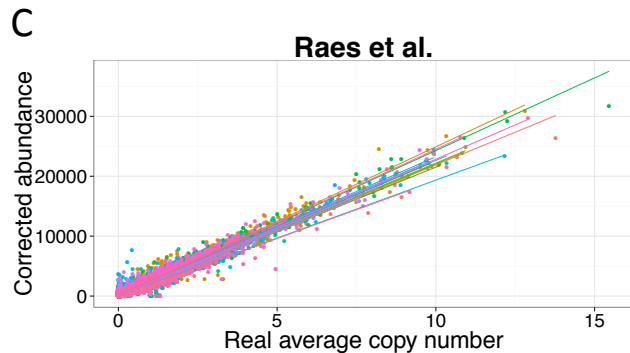

Supplement: Additional file 11: Figure S6. — Evaluation of different average genome size based normalization methods across 20 simulated metagenomic samples. Scatter plots are as in Figure 9 using the real average genome size (A), the average genome size estimated by GAAS (B), or the average genome size estimated by Raes et al. (C), for normalization. [file 13059_2015_610_MOESM11_ESM.pdf]
