# Supplementary material for: MUSiCC: a marker genes based framework for metagenomic normalization and accurate profiling of gene abundances in the microbiome
Source: Genome Biol. 2015 Mar 25;16(1):53. doi: 10.1186/s13059-015-0610-8 (PMC4391136; doi:10.1186/s13059-015-0610-8)
Supplement: Additional file 8: Figure S4. — The impact of MUSiCC on the discovery of differentially abundant genes between HMP stool samples and samples from other HMP body sites, including (A) supragingival plaque, (B) buccal mucosa, (C) anterior nares, (D) posterior fornix, and (E) retroauricular crease. Venn diagrams are defined as in Figure 6. [file 13059_2015_610_MOESM8_ESM.pdf]

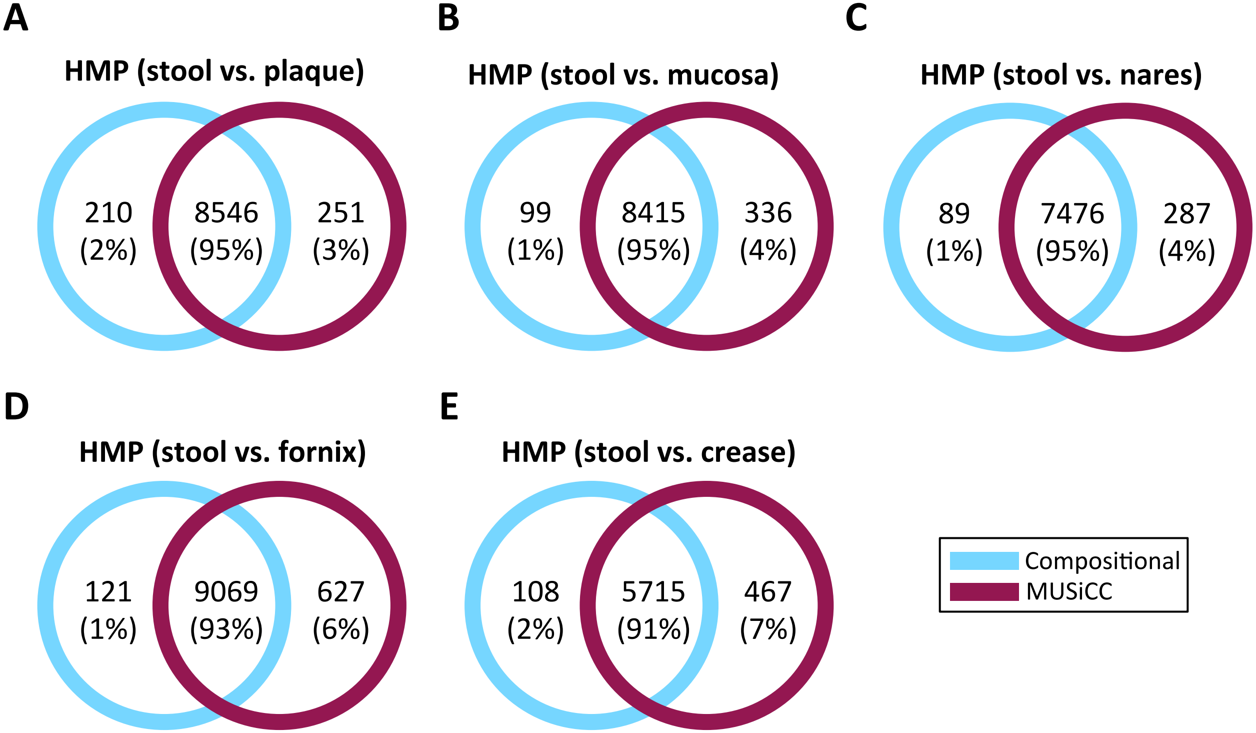
A
HMP (stool vs. plaque)
210 (2%)
8546 (95%)
251 (3%)
B
HMP (stool vs. mucosa)
99 (1%)
8415 (95%)
336 (4%)
C
HMP (stool vs. nares)
89 (1%)
7476 (95%)
287 (4%)
D
HMP (stool vs. fornix)
121 (1%)
9069 (93%)
627 (6%)
E
HMP (stool vs. crease)
108 (2%)
5715 (91%)
467 (7%)
Compositional
MUSiCC
